# Periostin and tenascin-C interaction promotes angiogenesis in ischemic proliferative retinopathy

**DOI:** 10.1038/s41598-020-66278-1

**Published:** 2020-06-09

**Authors:** Yuki Kubo, Keijiro Ishikawa, Kenichiro Mori, Yoshiyuki Kobayashi, Takahito Nakama, Mitsuru Arima, Shintaro Nakao, Toshio Hisatomi, Masatoshi Haruta, Koh-Hei Sonoda, Shigeo Yoshida

**Affiliations:** 1 Department of Ophthalmology, Kyushu University Graduate School of Medical Sciences, Fukuoka, Japan; 2 Department of Ophthalmology, Kurume University School of Medicine, Kurume, Japan

**Keywords:** Target identification, Molecular biology, Retinal diseases, Inflammation

## Abstract

Ischemic proliferative retinopathy (IPR), such as proliferative diabetic retinopathy (PDR), retinal vein occlusion and retinopathy of prematurity is a major cause of vision loss. Our previous studies demonstrated that periostin (PN) and tenascin-C (TNC) are involved in the pathogenesis of IPR. However, the interactive role of PN and TNC in angiogenesis associated with IPR remain unknown. We found significant correlation between concentrations of PN and TNC in PDR vitreous humor. mRNA and protein expression of PN and TNC were found in pre-retinal fibrovascular membranes excised from PDR patients. Interleukin-13 (IL-13) promoted mRNA and protein expression of PN and TNC, and co-immunoprecipitation assay revealed binding between PN and TNC in human microvascular endothelial cells (HRECs). IL-13 promoted angiogenic functions of HRECs. Single inhibition of PN or TNC and their dual inhibition by siRNA suppressed the up-regulated angiogenic functions. Pathological pre-retinal neovessels of oxygen-induced retinopathy (OIR) mice were attenuated in PN knock-out, TNC knock-out and dual knock-out mice compared to wild-type mice. Both *in vitro* and *in vivo*, PN inhibition had a stronger inhibitory effect on angiogenesis compared to TNC inhibition, and had a similar effect to dual inhibition of PN and TNC. Furthermore, PN knock-out mice showed scant TNC expression in pre-retinal neovessels of OIR retinas. Our findings suggest that interaction of PN and TNC facilitates pre-retinal angiogenesis, and PN is an effective therapeutic target for IPR such as PDR.

## Introduction

Ischemic proliferative retinopathy (IPR), including proliferative diabetic retinopathy (PDR), retinal vein occlusion and retinopathy prematurity is a major cause of visual loss and blindness^1^. Advanced IPR forms pre-retinal neovascularization and fibrovascular membrane (FVM) leading to vitreous hemorrhage and tractional retinal detachment. Recently, advancements in surgical techniques and pharmacological therapies have improved the visual prognosis of IPR. In particular, anti-vascular endothelial growth factor (VEGF) therapies prior to vitrectomy facilitates easier surgery due to their regressive effects on pre-retinal neovascularization^2,3^. On the other hand, several recent reports suggest that anti-VEGF therapy might have vision threatening adverse effects, such as thinning of the neurosensory retina after continuous injections of these drugs^4,5^, and intraocular fibrosis development that can lead to tractional retinal detachment^6–9^. Considering the above risks, development of a safe and effective therapeutic agent is required that can control fibrovascular proliferation.

To develop novel therapeutic strategies based on comprehensive understanding of the molecular mechanisms of fibrovascular proliferation associated with IPR, we performed a gene expression-profiling of FVMs with DNA microarray analysis. The results revealed that periostin (PN) and tenascin-C (TNC) were specifically expressed in FVMs^10^. PN and TNC are members of matricellular family of proteins that are defined by their ability to bind to both extracellular matrix and cell surface receptors with high expression in conditions involving development and tissue remodeling such as wound healing. Previous studies have shown that PN and TNC promote regeneration of heart tissue after myocardial infarction, cutaneous wound healing and tumorigenesis^11–16^.

Our group subsequently demonstrated that PN and TNC were highly up-regulated in the vitreous humor of patients with PDR and proliferative vitreoretinopathy, and promoted both retinal angiogenesis and fibrosis in the pathogenesis of vitreoretinal diseases^17–20^. Accumulated evidence suggest that ECM components including PN, TNC and fibronectin (FN) that is a well-characterized molecule, promotes physiological and pathological retinal angiogenesis^21–26^. These proteins interact with each other and form the ECM meshwork architecture during the process of scaffold remodeling in angiogenesis and fibrosis, such as bronchial asthma and intrahepatic cholangiocarcinoma^27–30^. However, interaction between PN and TNC in the pathogenesis of pre-retinal angiogenesis in eyes with IPR remains unknown.

The purpose of this study was to determine interaction between PN and TNC in eyes with IPR, especially PDR. We also investigated the inhibitory effects of PN and/or TNC inhibition on retinal angiogenesis in mice model of oxygen-induced retinopathy and *in vitro* human retinal microvascular endothelial cells (HRECs) to identify therapeutic targets to control fibrovascular proliferation in IPR such as PDR.

## Results

### Vitreous levels of PN, TNC and FN

**Figure 1:**
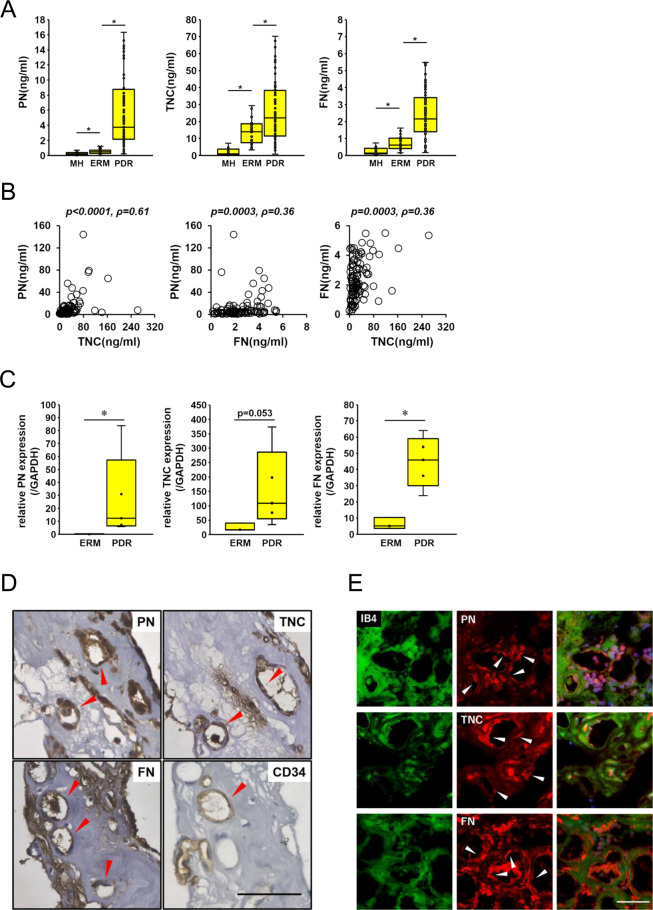
PN, TNC and FN are expressed in vitreous humor and FVMs from eyes with PDR patients. (**A**) Concentrations of PN, TNC and FN in the vitreous humor collected from eyes with macular hole (MH; n = 35), epiretinal membrane (ERM; n = 38) and proliferative diabetic retinopathy (PDR; n = 96). ^⋆^p < 0.005, Wilcoxon rank sum test. (**B**) Correlations among PN, TNC and FN in the vitreous humor of eyes with PDR (n = 96). Statistical significance was evaluated using Spearman’s rank correlation coefficient. (**C**) mRNA levels of PN, TNC and FN in ERMs (n = 3) and FVMs from eyes with PDR (n = 6) were assessed by qRT-PCR. All mRNA levels were normalized to GAPDH. *p < 0.05, Wilcoxon rank sum test. (**D**) Localization of PN, TNC and FN in FVMs. Red arrow heads indicate positive staining in the endothelium of neovessels. CD34 antibody was used as the positive control antibody staining the endothelium of neovessels. Hematoxylin and eosin procedure was performed as the counter stain. Scale bar = 50 μm. (**E**) Co-staining of PN, TNC and FN with IB4 in FVM sections. White arrow heads indicate positive staining of PN, TNC and FN in the endothelium of neovessels. FITC-conjugated IB4 was used for staining the endothelium of neovessels. Nuclei are stained blue. Scale bar = 50 μm.

To examine the correlation between PN, TNC and FN, we quantified concentrations of PN, TNC and FN in the vitreous humor obtained from patients with PDR and non-diabetic controls, macular hole (MH) and epiretinal membrane (ERM) by ELISA. The mean vitreous concentration of PN was significantly higher in PDR patients (10.80 ± 2.08 ng/mL) than those in MH and ERM patients (0.28 ± 0.05 ng/ml and 1.13 ± 0.62 ng/mL, respectively). The mean vitreous concentration of TNC was significantly higher in PDR patients (32.65 ± 3.84 ng/mL) than those in MH and ERM patients (2.64 ± 0.58 ng/mL and 16.59 ± 2.59 ng/mL, respectively). The mean vitreous concentration of FN was also significantly higher in PDR patients (2.43 ± 0.14 ng/mL) than those in MH and ERM patients (0.33 ± 0.07 ng/mL and 0.84 ± 0.11 ng/mL, respectively). The mean concentrations of PN, TNC and FN in ERM patients were significantly elevated than those of MH patients (Fig. 1A). In addition, we examined the correlation among the various vitreous concentrations of PN, TNC and FN in 96 eyes of PDR patients. There was a significant correlation between PN and TNC (ρ = 0.61, p < 0.0001) as previously reported^20^. Significant correlations were also found between PN and FN (ρ = 0.36, p = 0.0003), and between TNC and FN (ρ = 0.36, p = 0.0003) (Fig. 1B).

### mRNA expression of PN, TNC and FN in FVMs from patients with PDR

To determine whether these three proteins are associated with formation of FVMs, we quantified mRNA expression levels of PN, TNC and FN by real-time qRT-PCR on the RNA extracted from FVMs in PDR patients and non-vascularized fibrous membranes in ERMs. The mean mRNA expression levels of PN and FN in FVMs from PDR patients were significantly higher than those from ERM patients. The mean TNC mRNA levels in FVMs were higher than those in ERMs, albeit with no significance (p = 0.053) (Fig. 1C).

### Localizations of PN, TNC and FN in FVMs

Since the mRNA of PN, TNC and FN were all expressed in FVMs excised from PDR retinas, we performed immunohistochemical and immunofluorescent staining of serial FVM sections to examine localizations of PN, TNC and FN proteins. Positive staining of these three proteins were seen around tubular structures in FVMs and these structures were also stained with CD34 and isolectin-B4 (IB4) known as vascular endothelial markers (Fig. 1D,E). These findings suggested that PN, TNC and FN co-localized in the endothelium of neovessels in PDR-FVMs.

### mRNA and protein expression of PN, TNC and FN in HRECs stimulated by IL-13

**Figure 2:**
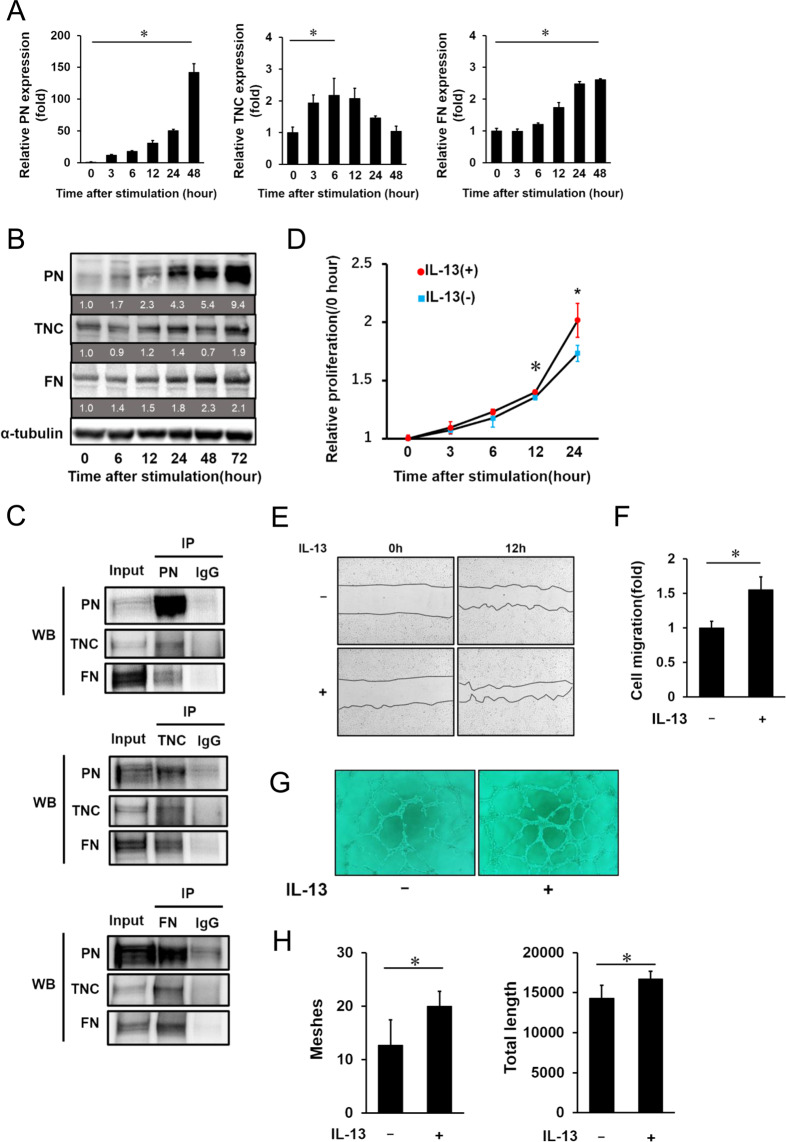
IL-13 induces PN, TNC and FN expressions and promotes proliferation, migration and tube formation of HRECs. Expression of PN, TNC and FN mRNA (**A**) and protein (**B**) in HRECs stimulated by IL-13 (10 ng/ml). (**A**) PN, TNC and FN mRNA expression after IL-13 stimulation at different time points determined by qRT-PCR. All mRNA levels were normalized to GAPDH mRNA expression. Relative mRNA levels at 3, 6, 12, 24 and 48 h after IL-13 stimulation are shown as fold changes. Error bars represent standard deviations. *p < 0.05, Wilcoxon rank sum test, n = 4/group. (**B**) Western blot analysis of PN, TNC and FN in the cell lysates of HRECs after stimulation by IL-13 at different time points. α-tubulin was used as a reference protein. Protein expression levels relative to 0 h were labeled under each corresponding band. (**C**) Co-immunoprecipitation of cell lysates from HRECs stimulated by IL-13. The cell lysates were immunoprecipitated with IgG, anti-PN antibodies, anti-TNC antibodies and anti-FN antibodies. The precipitates were analyzed by western blotting with anti-PN, anti-TNC and anti-FN antibodies. Full-length gel images of Fig. 2B,C are displayed in Supplementary Figure S1, and graph images showing quantification of signal intensity of the bands are placed in Supplementary Figure S2. Role of IL-13 in cell proliferation (**D**), cell migration (**E**,**F**) and tube formation (**G**,**H**) of HRECs. (**D**) Relative proliferation rate of HRECs treated with IL-13 is shown as growth curves. Error bars represent standard deviations. ^⋆^p < 0.01, *p < 0.05, Wilcoxon rank sum test, n = 6/group. (**E**) Representative images of scratch wound healing assay in HRECs treated with or without IL-13. The left images show scratched areas before IL-13 stimulation and the right images show the same areas at 12 h post stimulation. (**F**) Cell migration was assayed by calculating areas compensated by migrated HRECs. The data of HRECs with IL-13 stimulation group is shown as fold changes. Error bars represent standard deviations. *p < 0.05, Wilcoxon rank sum test, n = 4/group. (**G**) Representative images showing HRECs tube formation with or without IL-13 stimulation. (**H**) The graphs show the quantification of meshes and total length of HRECs with IL-13 treatment in the tube formation assay. Error bars represent standard deviations. *p < 0.05, Wilcoxon rank sum test, n = 6/group.

Since localization of PN, TNC and FN were seen in the vascular endothelium of FVMs, we performed *in vitro* experiments using human retinal microvascular endothelial cells (HRECs). Our previous study reporting elevated vitreous concentration of interleukin-13 (IL-13) in PDR patients^31^ led us to hypothesize that IL-13 induces synthesis and secretion of PN, TNC and FN in HRECs. First, we stimulated HRECs with IL-13 and quantified mRNA levels of those proteins by real-time qRT-PCR. mRNA expression of PN was up-regulated at 3 h and increased up to 48 h after IL-13 stimulation. The peak mRNA level of PN increased approximately 140 folds compared to that of non-treated controls. mRNA expression of TNC up-regulation started at 3 h, but the expression level peaked at 6 to 12 h and was down-regulated 48 h after the stimulation. We found that the peak expression level of TNC mRNA was increased 2 to 3 folds compared to the control samples. mRNA level of FN up-regulation started at 6 h and increased up to 48 h after the stimulation. The FN mRNA level 48 h after the stimulation was two to three times higher than the level of non-treated controls (Fig. 2A).

Next, we examined protein expression of HRECs stimulated by IL-13. Western blotting analysis showed that protein expression of PN and FN gradually increased after IL-13 stimulation, while TNC expression transiently decreased 48 h after IL-13 stimulation (Fig. 2B).

### Co-immunoprecipitation of PN, TNC and FN in HRECs stimulated by IL-13

Since the correlation among PN, TNC and FN concentrations in the vitreous humors with PDR patients were seen, we next conducted co-immunoprecipitation (co-IP) using lysates from HRECs stimulated by IL-13 to test for protein interaction among these three proteins. TNC and FN were observed to be co-immunoprecipitated with PN. In addition, we found that PN and FN co-immunoprecipitated with TNC and PN and TNC co-immunoprecipitated with FN (Fig. 2C, Supplementary Figure S2). These results suggested that PN, TNC and FN directly bound to each other or were possibly included in the protein complex extracted from the lysates of HRECs stimulated by IL-13.

### Induction of cell proliferation, cell migration and tube formation in HRECs stimulated by IL-13

Next, we investigated the effects of IL-13 on the cellular functions associated with fibrovascular proliferation, such as cell proliferation, cell migration and tube formation using HRECs. We first conducted a cell proliferation assay using Cell Counting Kit-8. IL-13 stimulation for 12 and 24 h significantly promoted cell proliferation of HRECs (Fig. 2D). We next studied whether IL-13 affected cell migration of HRECs. Our scratch wound healing assay showed 50% increase in compensation of scratch gap by HRECs stimulated by IL-13 compared to HRECs without stimulation (Fig. 2E,F). Lastly, we demonstrated tube formation assay using a Matrigel matrix. Under stimulation of IL-13, both meshes and total lengths of tubes composed of HRECs were increased (Figure G,H). According to these findings, IL-13 enhanced cell proliferation and cell migration of HRECs and promoted subsequent vascular branch formation and expansion.

### Localization of PN, TNC and FN in retinas of murine oxygen-induced retinopathy models

To further clarify the interactive roles of PN, TNC and FN in retinal neovascularization, we used a mouse model of oxygen-induced retinopathy (OIR), a well-established *in vivo* model of hypoxia-induced vascular proliferation.

**Figure 3:**
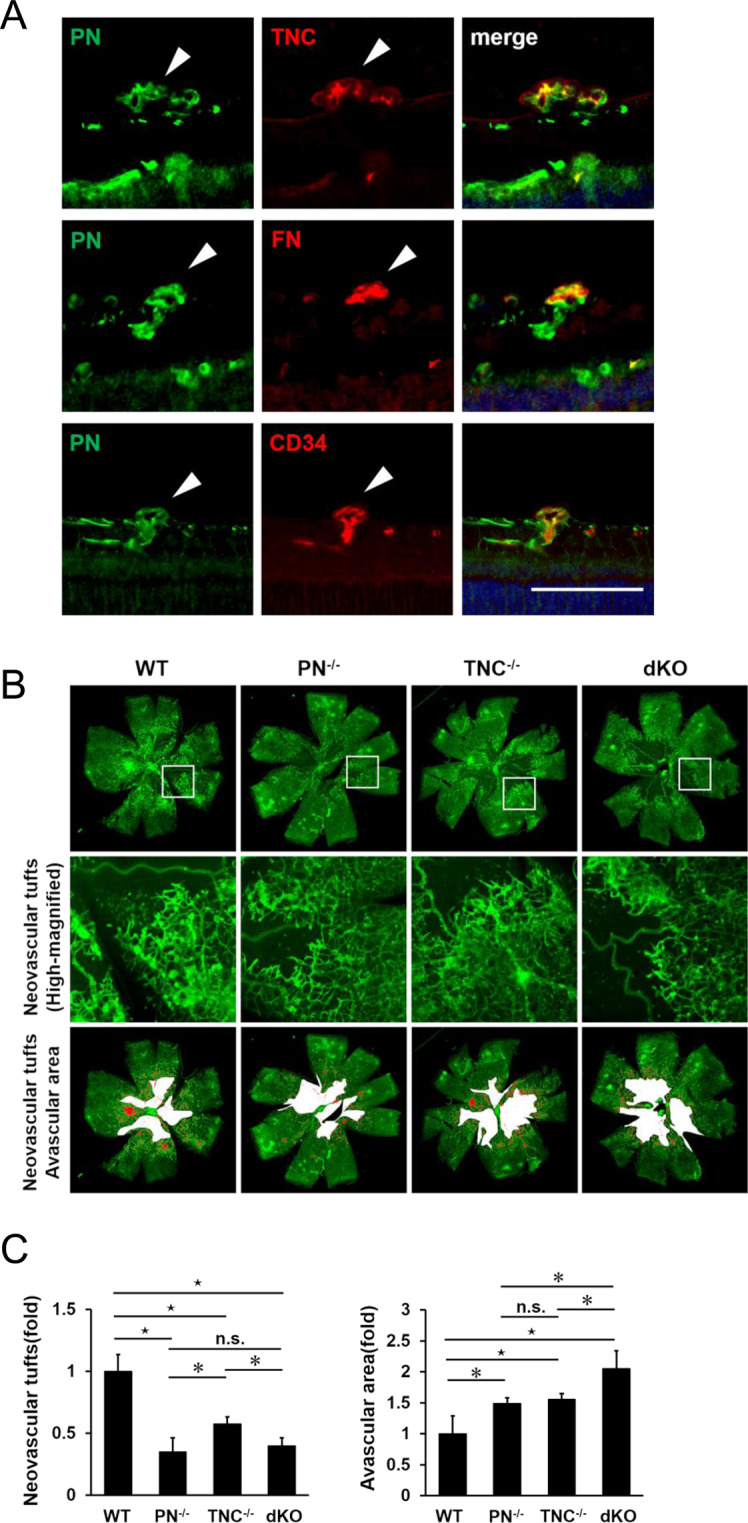
Genetic ablation of PN and/or TNC reduces physiological revascularization and pathological neovascularization in OIR retinas. (**A**) Immunofluorescence staining for PN, TNC, FN and CD34 (a marker for vascular endothelial cells) in retinal sections from WT OIR mice of P17. White arrow heads indicate neovascular tufts. Nuclei are stained blue. Scale bar = 50 µm. (**B**) Flat mounts of P17 OIR retinas in WT, PN^−/−^, TNC^−/−^, PN^−/−^TNC^−/−^ (dKO) mice. Top panels show images stained with FITC-conjugated IB4. Middle panels show highly magnified images of the white rectangular boxes in top panels. Lower panels show pathological neovascular tufts (red colored) and avascular areas (white colored) measured for quantification. (**C**) Quantification of avascular areas and neovascular tufts of OIR retinas in WT, PN^−/−^, TNC^−/−^ and dKO mice. Fold changes of avascular areas and neovascular tufts of OIR retinas in PN^−/−^, TNC^−/−^ and dKO mice compared to those in WT mice. The right graph shows relative fold of avascular areas and the left graph shows relative fold of neovascular tufts compared to WT mice. ^⋆^p < 0.01, *p < 0.05, Wilcoxon rank sum test, n = 6/group.

First, to investigate whether these three proteins are expressed in this model, we performed immunofluorescence staining of sections in wild-type (WT) OIR retinas at P17 when pathological neovascularization is highly promoted. PN expression was seen in pre-retinal neovascular tufts (NVTs) and the inner retinal vasculature which NVTs branched from. Co-localization of PN with TNC, FN and CD34, a vascular endothelial cell marker, were observed especially in NVTs. This result suggested that these three proteins were localized in proliferative vascular endothelium of OIR retinas (Fig. 3A). These findings were consistent with the observation showing expression of the three proteins in neovascular vessels in FVMs of PDR patients.

### Inhibitory effect of PN and/or TNC deletion on hypoxia-induced angiogenesis in OIR mice retinas

Next, to investigate the roles of PN and TNC in hypoxia-induced physiological and pathological angiogenesis, retinal flat mounts of PN knockout (PN^−/−^), TNC knockout (TNC^−/−^) and PN^−/−^TNC^−/−^ (double knockout: dKO) OIR mice were stained with IB4 (Fig. 3B). Avascular areas (AVs) of all the PN^−/−^, TNC^−/−^ and dKO mice were significantly larger than those of WT littermates. Significant difference of the mean AVs was not seen between PN^−/−^ and TNC^−/−^ mice (Fig. 3C). Furthermore, we performed IB4 staining in the retinal flat mounts of these four strains of mice at postnatal day 5 to investigate the normal retinal vascular development, which could affect the subsequent pathogenesis of OIR. There was no obvious difference in the vasculatures among these four strains (Supplementary Figure S3). These observations suggest that genetic deletion of PN and TNC could inhibit hypoxia-induced intra-retinal angiogenesis and their dual deletion had an additive inhibitory effect. The mean areas of NVTs in P17 PN^−/−^, TNC^−/−^ and dKO OIR mice were significantly smaller than those of WT OIR mice. The mean area of PN^−/−^ mice was significantly smaller compared to that of TNC^−/−^ mice but not significantly different from that of dKO mice. These findings suggest that genetic deletion of PN and TNC could inhibit hypoxia-induced pre-retinal angiogenesis. The inhibitory effect of PN deletion was stronger than that of TNC deletion, and was similar to that of dual deletion.

### Inhibitory effects of PN and/or TNC on cellular functions of HRECs

**Figure 4:**
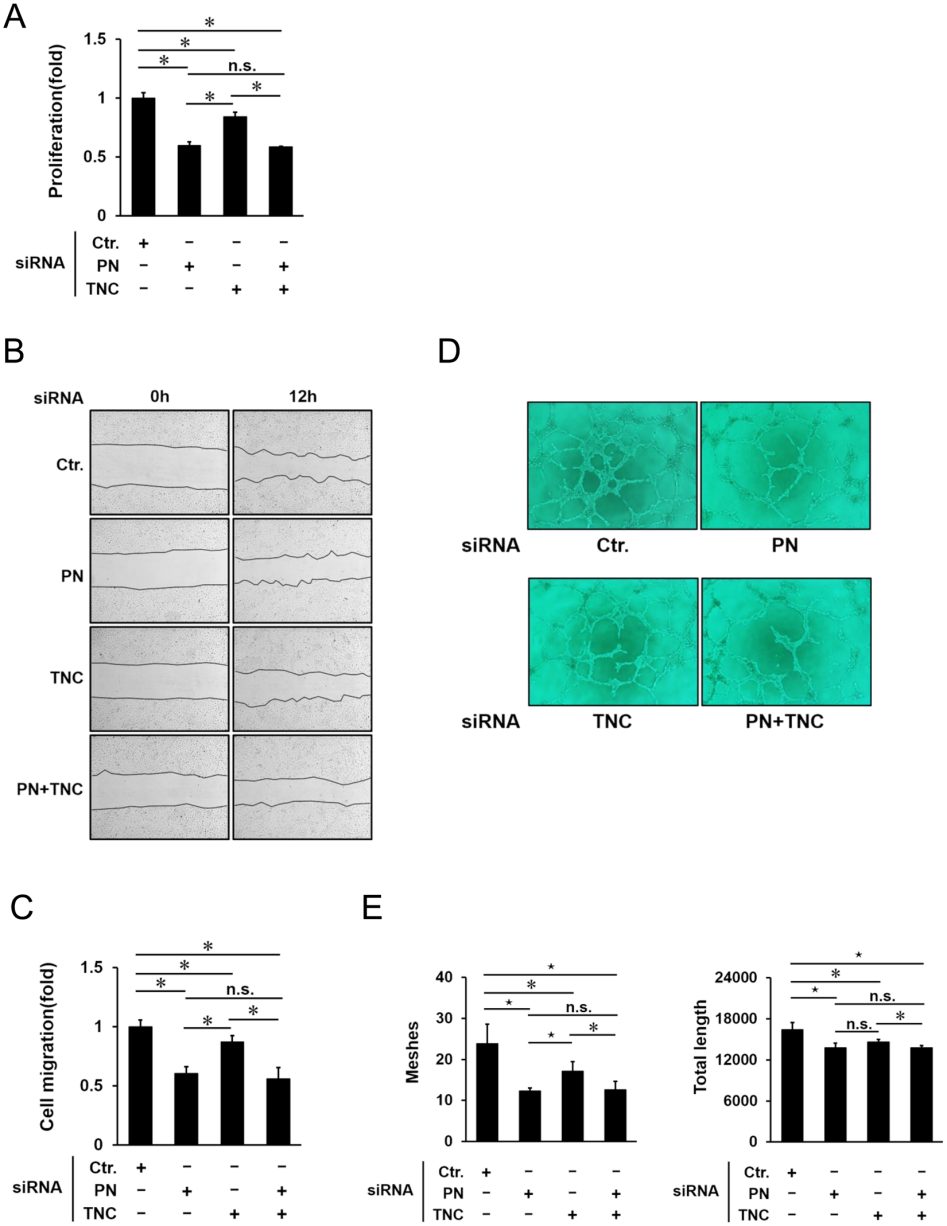
Inhibition of PN and/or TNC suppresses angiogenic functions; cell proliferation, cell migration and tube formation. (**A**) Cell proliferation was assessed by CCK-8 assay. Effects of PN, TNC and their dual inhibition by siRNA on the cells treated with IL-13 for 24 h. Proliferation rates of HRECs are shown as fold changes. Error bars represent standard deviations. *p < 0.05, Wilcoxon rank sum test, n = 6/group. (**B**) Cell migration was assessed by scratch wound healing assay. Representative images of HRECs treated with PN, TNC and their dual inhibition by siRNA. The left images show the scratched cells at 0 h (Left) and 12 h (Right) after IL-13 stimulation. (**C**) Effects of PN, TNC and their dual inhibition by siRNA on cell migration were quantified by measuring the areas of migrated cells at 12 h after IL-13 treatment. The data are shown as fold changes. Error bars represent standard deviations. *p < 0.05, Wilcoxon rank sum test, n = 4/group. (**D**) Representative images of HRECs treated with PN, TNC and their dual inhibition by siRNA in Matrigel tube formation assay. (**E**) Data show the quantification of the total numbers of meshes and total length. Error bars represent standard deviations. ^⋆^p < 0.01, *p < 0.05, Wilcoxon rank sum test, n = 6/group.

Since IL-13 promoted mRNA and protein expression of PN and TNC from HRECs, and also promoted cell proliferation, cell migration and tube formation of HRECs, and genetic ablation of PN and TNC inhibited physiological and pathological angiogenesis *in vivo*, we next postulated that PN and TNC secreted from HRECs promoted their cell functions such as proliferation, migration and tube formation. To test this hypothesis, we examined inhibitory effects of PN and/or TNC inhibition on cellular functions of HRECs promoted by IL-13. Cell proliferation of HRECs was inhibited by both PN and TNC siRNA, and PN siRNA had a higher inhibitory effect than TNC siRNA. On the other hand, no significant difference between single administration of PN siRNA and combined administration of PN siRNA and TNC siRNA was seen (Fig. 4A). Next, scratch wound healing was also inhibited by both siRNA, and PN siRNA was more effective than TNC siRNA. As additive effect of PN siRNA and TNC siRNA was not shown in this assay (Fig. 4B,C). With regard to tube formation, the numbers of meshes and total lengths were decreased by both siRNA, and PN siRNA was more effective than TNC siRNA to decrease the numbers of meshes. Combined administration of PN and TNC siRNA did not show additive inhibitory effect on tube formation (Fig. 3D,E). Although mRNA and protein expression levels of PN and TNC from HRECs stimulated by IL-13 were different, these results suggested that both PN and TNC contributed to angiogenic functions of HRECs stimulated by IL-13. Furthermore, consistent with the *in vivo* study above, single inhibition of PN had a similar inhibitory effect to dual inhibition of PN and TNC on cellular angiogenic function of HRECs.

Hypoxia inducible factor (HIF) is one of the important molecules regulating hypoxia-induced angiogenesis^32,33^. To check if HIF pathway is associated with PN and TNC inducible angiogenesis, we investigated expression changes of HIF-associated genes induced by IL-13 stimulation and inhibition of PN or TNC. The results showed that IL-13 stimulation could upregulate mRNA expressions of HIF2α and VEGFA, which were not affected by treatment with PN or TNC siRNA (Supplementary Figure S4, and primers are listed in Supplementary Table). This suggests that the facilitatory roles of PN and TNC in hypoxia-induced angiogenesis could be independent of HIF-related pathways.

### Diminished TNC expression in pre-retinal neovascular tufts in PN^−/−^ OIR mice

**Figure 5:**
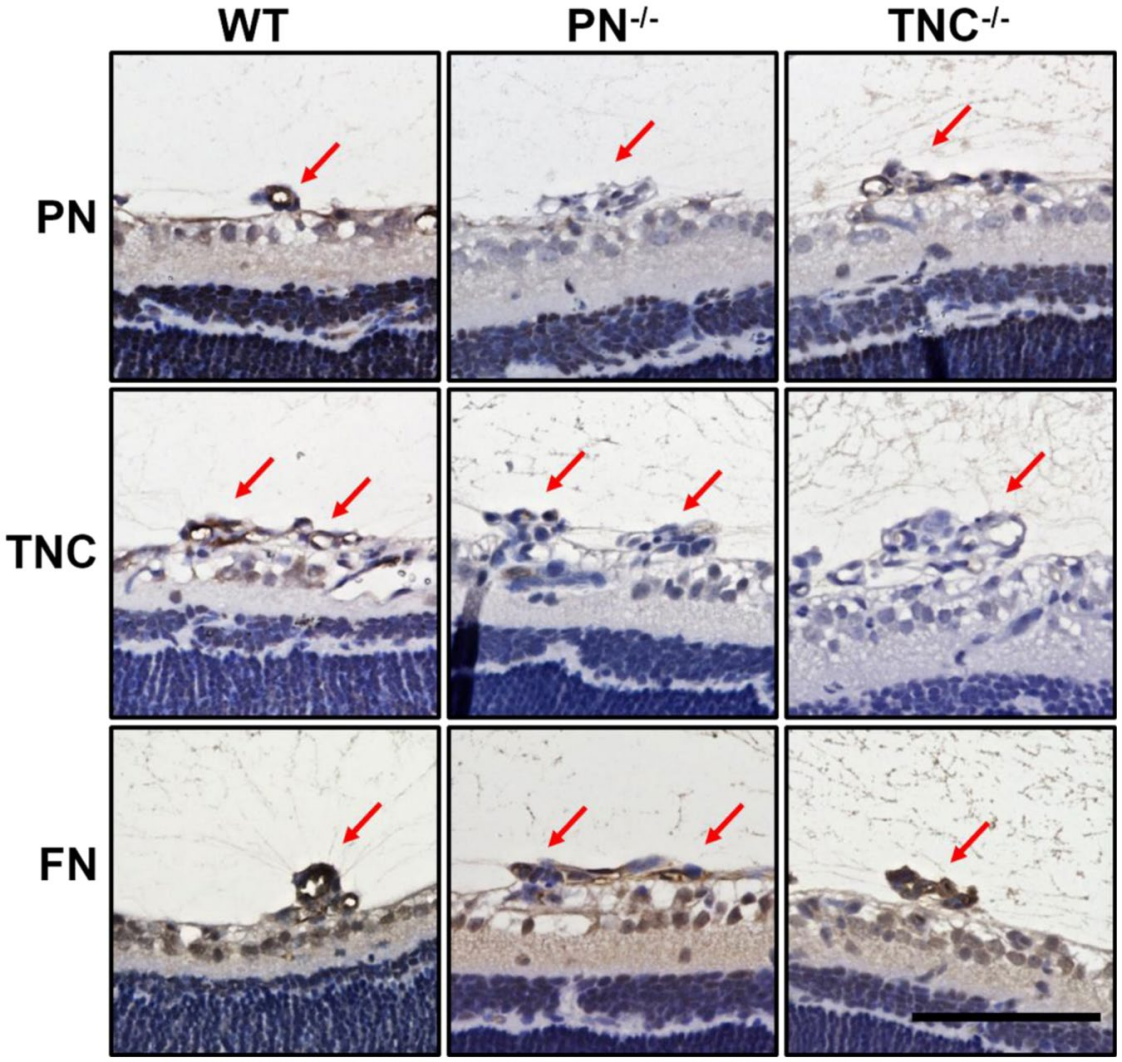
Immunohistochemical staining for PN and TNC in OIR retina sections from WT, PN^−/−^ and TNC^−/−^ mice. Left panels show the positive staining of PN, TNC and FN antibodies in neovascular tufts of WT retinas. Weak staining of TNC in the tufts of PN^−/−^ retina (central panel). Significant staining of PN is shown in the tufts of TNC^−/−^ retina (upper right panel). Hematoxylin and eosin procedure was performed as the counter stain. Red arrows indicate neovascular tufts. Scale bar = 50 µm.

Since TNC inhibition did not have an additive inhibitory effect on angiogenesis where PN was inhibited both *in vitro* and *in vivo*, we sought to identify the causative mechanisms. First, we hypothesized that PN would be an upstream regulator of TNC and sole PN inhibition could thus down-regulate TNC expression resulting in sufficient suppressive effect as compared to dual PN and TNC inhibition. For verification, we checked TNC mRNA expression after PN siRNA transfection in HRECs, and found no significant change (Supplementary Figure S5). Since previous studies reported that PN functions as a bridge for the interaction between TNC and the ECM including FN in the meshwork^29^, we next hypothesized that PN deletion would block TNC aggregation in NVTs. Immunohistochemical staining of the NVTs revealed that TNC signals were barely seen in PN^−/−^ OIR mice while significant signals were apparent in WT OIR mice. On the other hand, PN signal in the NVTs in TNC^−/−^ OIR mice were similarly seen to those in WT OIR mice. FN signals were significant in PN^−/−^ and TNC^−/−^ OIR mice as seen in WT mice (Fig. 5).

These findings suggested that PN could aggregate with TNC to the ECM in the lesions of NVTs and therefore, sole PN inhibition had an inhibitory effect on angiogenesis equivalent to that of dual PN and TNC inhibition.

## Discussion

This study indicates for the first time that PN, TNC and FN interact with each other to assemble into the protein complex that can promote angiogenic functions of vascular endothelial cells in pre-retinal neovascularization of IPR. Furthermore, we demonstrate that PN inhibition is an effective therapeutic strategy in hypoxia-induced retinal neovascularization model.

Previous studies demonstrated potential in promoting retinal neovascularization for PN, TNC and FN respectively; however, their interactive roles remained unknown. Our data showed significant correlation among vitreous levels of PN, TNC and FN in patients with PDR and co-localization of these proteins in neovascular vessels in human FVMs and mice OIR retinas. Moreover, *in vitro* co-immunoprecipitation assay revealed evidence for the existence of those proteins complex in vascular endothelial cells. This observation is in line with the previous report suggesting that multiple ECM components form a complex meshwork serving as the scaffold of cell proliferation and migration in pathophysiological conditions such as tissue development and regeneration, tumorigenesis and fibrosis^28^. Taken together, in the pathogenesis of IPR, PN, TNC and FN interact with each other to form an ECM complex that can become a scaffold for promoting angiogenesis in vascular endothelial cells.

IL-13 is an important type 2 T helper (Th2) cytokine controlling biological functions in tissue remodeling such as angiogenesis and fibrosis^34–37^. We previously found IL-13 up-regulation in the vitreous humors of PDR patients and OIR mice retinas, which suggests that ischemic injury induces IL-13 expression and secretion in the retina^19,31^. Meanwhile, after central nervous system (CNS) injury by stroke, a shift towards Th2 response and downregulation of Th1 response play a role in tissue remodeling during CNS wound healing response^38^. These imply that Th cells shift towards Th2 cells that are able to produce IL-13 in the ischemic retina. Our data showed that IL-13 induces PN, TNC and FN expression and secretion from HRECs and promotes their angiogenic functions including as cell proliferation, migration and tube formation. These suggest that Th2 cells infiltrating the ischemic retina can secrete lL-13 that act on retinal vascular endothelial cells to produce PN, TNC and FN, which result in retinal neovascularization in the pathogenesis of IPR.

Our results demonstrated that deletion of PN and/or TNC inhibited pre-retinal pathological neovascularization (NV) and intra-retinal physiological revascularization in OIR retina. The inhibitory effect of PN deletion on pre-retinal NV was greater than TNC deletion, but was comparable to the dual deletion of PN and TNC. Consistent with this result, PN inhibition had a stronger inhibitory effect on angiogenic functions of HRECs *in vitro* than TNC inhibition, while it had similar effects as their dual inhibition. Diminished TNC expression in the neovascular tufts in PN^−/−^ OIR mice indicates that PN inhibition could block TNC aggregation in the lesions. In contrast, PN expression in the lesion was not affected in TNC^−/−^ OIR mice. These results suggest that PN promotes incorporation of TNC into the ECM complex in the lesions, but not vice versa. Kii *et al*. previously demonstrated that PN possesses adjacent domains that bind to TNC and the other ECM proteins functioning as a bridge between TNC and the ECM^29^. Taken together, PN plays a crucial role in forming an ECM complex meshwork on which cellular signaling is activated during tissue remodeling processes including angiogenesis. On the other hand, PN deletion had a similar inhibitory effect on intra-retinal revascularization to TNC depletion, and the dual deletion of PN and TNC had an additive inhibitory effect. Previous reports suggested that TNC from vascular smooth muscle cells (VSMCs) act in a paracrine fashion to stimulate vascular endothelial cells to promote physiological and pathological angiogenesis^20,39^. Since VSMCs cover intra-retinal neovessels but are absent in the surroundings of pre-retinal neovessels^40^, intra-retinal revascularization can be more susceptible to TNC secreted from VSMCs than pre-retinal NV. Additionally, our *in vitro* data showed TNC up-regulation in vascular endothelial cells after IL-13 stimulation and this increased expression was inhibited by TNC siRNA; however, it was not as striking as the PN expression change and the effect of PN siRNA (Supplementary Figure S6) . These may reflect our observation that TNC inhibition had less impact on pre-retinal NV while it demonstrated an equivalent effect on intra-retinal revascularization as compared to PN inhibition.

Generally, retinal hypoxia triggers both physiological and pathological angiogenesis. Normal retinal vascular development can be tightly regulated with temporal and spatial pattern under a balance maintained between oxygen supply and demand in avascular areas of peripheral retinas. By contrast in avascular areas of OIR retinas, disorganized pathological angiogenesis can occur where an oxygen undersupply might exist^41,42^. Genetic depletion of PN and/or TNC did not affect normal retinal vascular development at postnatal day 5. Given that PN and TNC are identified as molecules specifically expressed in pathological fibrovascular tissue, PN and TNC might be disease-specific molecules responsible for pathological angiogenesis in the oxygen undersupply conditions.

**Figure 6:**
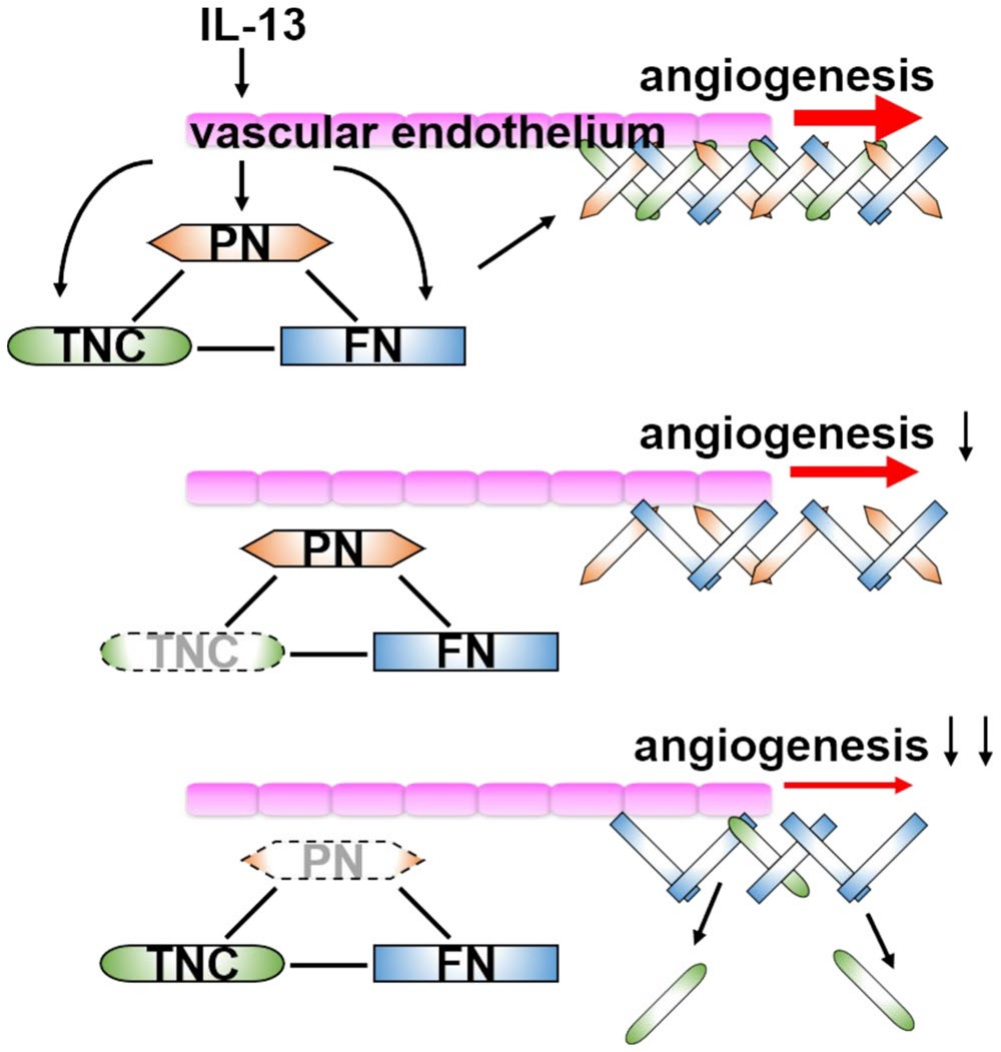
Hypothesis scheme of pre-retinal angiogenesis promoted by the ECM interaction and the inhibitory effect of PN and TNC on the angiogenesis. IL-13 promotes secretion of PN, TNC and FN from the vascular endothelium. These matricellular proteins form the ECM meshwork and this meshwork architecture promotes angiogenesis through activating angiogenic receptors such as integrins and functioning as scaffolds of cell proliferation, migration and tube formation. Inhibition of TNC causes a mild inhibitory effect on angiogenesis due to lack of only TNC. On the other hand, inhibition of PN significantly attenuated angiogenesis because lack of PN also causes scant TNC deposition to the ECM meshwork.

The results from this study propose that PN, TNC and FN are secreted from retinal microvascular endothelial cells by IL-13 stimulation to construct an ECM meshwork. The meshwork architecture can serve as a scaffold on which vascular endothelial cells promote angiogenic functions. As PN inhibition is able to block TNC aggregation in the neovessels, single PN inhibition can exert anti-angiogenic properties as effective as dual inhibition of PN and TNC (Fig. 6).

Multiple inhibition of matricellular proteins including TNC and FN could possibly interfere physiological intra-retinal revascularization. As single PN blockade could suppress pre-retinal pathological NV efficiently, PN is an attractive therapeutic target among matricellular proteins. Moreover, our previous studies revealed PN as a pivotal molecule for pre-retinal and subretinal fibrous membrane formation causing tractional retinal detachment^18,43^. Although the Diabetic Retinopathy Clinical Research Network (DRCR.net) Protocol S trial demonstrated that anti-VEGF therapy for PDR can be effective^44^, and that frequent use of anti-VEGF drugs can resolve diabetic macular edema and exudative age-related macular degeneration (AMD)^45–47^, some studies reported that anti-VEGF therapy is associated with fibrosis development in PDR and AMD patients^6–9^. Since PN inhibition has the potential to control both pathological NV and retinal fibrosis, PN blockade would be a promising therapeutic strategy for IPR such as PDR.

## Materials and methods

### Human specimens

This study was approved by the Ethics Committees of Kyushu University Hospital, and we handled human specimens in accordance with the Declaration of Helsinki. Vitreous humor and epiretinal membranes were obtained from patients who underwent pars plana vitrectomy following informed consent.

FVMs were collected from 6 eyes of 6 patients with PDR, where one proceeded to undergo an immunostaining procedure and the others underwent RNA extraction. Non-fibrovascular epiretinal membrane samples were collected from 3 eyes of 3 patients with idiopathic ERM. Vitreous samples were collected from patients with PDR (96 eyes of 87 patients, age; 59.2 ± 12.1, 62 men and 25 women), macular hole (MH, 35 eyes of 35 patients, age; 60.7 ± 6.7, 19 men and 16 women) and ERM (38 eyes of 37 patients, age; 67.3 ± 8.7, 16 men and 21 women), respectively. None of the patients in MH and ERM group had diabetes mellitus. Vitreous samples were aspirated at the beginning of vitrectomy then immediately transferred to sterile tubes. The samples were centrifuged for 10 min at 1630 × g at 4 °C, and the supernatant stored at −80 °C until analyses.

### ELISA

Protein concentrations of PN, TNC and FN were measured by ELISA kits (Supplementary Table) in accordance with the manufacturer’s instructions. Absorbance was measured to determine the concentration of PN, TNC and TNC at a wavelength of 450 nm by a micro plate reader (EnSpire; PerkinElmer).

### Real-time quantitative reverse transcription PCR

Total RNA was extracted from homogenized human FVMs, ERMs and cultured HRECs by Magtration System 6GC and MagDEA RNA kit (A1002 and E7004; Precision System Science). Extracted RNA was reverse-transcripted with a Transcriptor First Strand cDNA Synthesis Kit (#04379012001; Roche) and GeneAmp PCR System 9700 (#9700; Applied Biosystems). Gene expression of the cDNA was quantified using TaqMan probe for detection of TNC or SYBR Green (RR430A; TAKARA) and primers for detection of PN and FN. The amplified fluorescence signal was analyzed using a LightCycler 96 (#05815916001; Roche). TaqMan probe and the primer sequences used are indicated in Supplementary Table.

### Immunohistochemistry and immunofluorescence

FVMs and OIR mice retinas were embedded in paraffin and sliced to 3 µm sections. After removal of the paraffin, we rehydrated, blocked and incubated the sections with primary antibodies (Supplementary Table) for 24 h at 4 °C. For immunohistochemistry, the bound antibodies were visualized by an avidin-biotin peroxidase procedure with 3,3′-diaminobenzidine (415172; Nichirei) as the substrate. Counterstaining of nuclei was performed using hematoxylin and eosin procedure. For immunofluorescence analysis, the fluorescence-conjugate secondary antibodies (Supplementary Table) were added for an hour at room temperature and subsequent staining of fluorescein-labeled isolectin-B4 (IB4) (FL-1201; VECTOR laboratories) for 90 minutes at room temperature was performed in FVM sections. Nuclei were counterstained with Hoechst33342 (H3570; Invitrogen). The specimens were mounted in mounting medium (TA030FM; Thermo Fisher) and examined with a microscope (BZ-X800: Keyence).

### Cell culture

Primary HRECs were purchased from Cell Systems (ACBRI 181) and used for the *in vitro* studies. Cells were cultured in Complete Medium Kit With Serum and Cultureboost-R (4Z0-500-R; Cell Systems) at 37 °C in 5% CO_2_. Cells between the fifth to seventh passage were used for experiments.

### Western blotting

HRECs were seeded onto a collagen coated 6-well dish and cultured to 80% confluence. These cells were incubated with Endothelial Cell Growth Medium-2 (EGM-2) medium (CC3162; Lonza) containing 1% FBS and 10 ng/ml recombinant human IL-13 (213-ILB-005; R&D systems). Subsequently, we washed these cells with PBS and added 100 µl of ice cold 0.1% SDS containing lysis buffer (composed of 25 mM Tris-HCL, 150 mM NaCl and 1% Triton X-100) with the protease and phosphatase Inhibitor (#78440; Thermo Fisher). After incubation on ice for 15 minutes, the lysates were centrifuged at 15000 rpm for 15 minutes at 4 °C. The supernatant was collected and boiled with sample buffer that was composed of Laemli buffer (AAJ61337AC; Thermo Fisher) supplemented 5% 2-mercaptoethanol (#516732; Sigma-Aldrich) at 95 °C. The protein samples were separated on a NuPAGE 3–8% Tris-Acetate gel (EA0375; Invitrogen). Blotting membranes were incubated with blocking buffer (#37543; Thermo Fisher) for 30 minutes and antibodies against PN, TNC and FN (Supplementary Table) for an hour at room temperature. After incubation with horseradish peroxidase (HRP) conjugated secondary antibodies (Supplementary Table), the membranes were incubated with a luminescent substrate (#34095; Thermo Fisher) and luminescence was detected using EZ-Capture MG (AE-9300; ATTO).

### Immunoprecipitation

HRECs were seeded onto a collagen coated 10 cm dish and cultured to 80% confluence. These cells were incubated for 72 h with EGM-2 medium containing 1% FBS and 10 ng/ml IL-13. Cell lysates were collected using 400 µl of lysis buffer without SDS and the supernatant was incubated with primary antibodies against PN, TNC and FN (Supplementary Table) using an end-over-end shaker at 4 °C. After over-night incubation, the protein/antibody complex was mixed with pre-cleaning agarose beads (#17061801; GE healthcare) and extra incubation for 2 h at 4 °C was performed. Then, the samples were boiled with the sample buffer and underwent western blotting.

### Cell proliferation assay

Cell proliferation was measured by Cell Counting Kit-8 (CCK-8) assay (CK04-11, Dojindo) according to the manufacturer’s instructions. Briefly, For describing growth curve of HRECs, We seeded HRECs onto a collagen coated 10 cm dish and incubated to 80–90% confluency. Subsequently, these cells were splitted to collagen coated 96-well plate at 5.0 × 10^3^ cells/well in EGM-2 medium containing 1% FBS and human recombinant IL-13 at 10 ng/ml. After 3, 6, 12 and 24 h of incubation, we added 10 μl of the CCK-8 solution to each well of the plate. For the experiment of PN and/or TNC knock down, HRECs were seeded onto a collagen coated 6-well plate at 5.0 × 10^4^ cells/well and transfected with control, PN and TNC siRNA. After 24 h of incubation, we seeded these cells onto a collagen coated 96-well plate at 5.0 × 10^3^ cells/well in EGM-2 medium containing 1% FBS and human recombinant IL-13 at 10 ng/ml for 24 h. Subsequently, we added 10 μl of the CCK-8 solution to each well of the plate. In both experiments, after 2 h of incubation with CCK-8, we measured the absorbance at a wavelength of 450 nm using the microplate reader.

### Scratch wound healing assay

Scratch wound healing assay was conducted using CELL Scratcher scratch guide (9220-002; IWAKI) and 1000 µl pipette tips. Briefly, HRECs were seeded onto a collagen coated 24-well at 1.0 × 10^4^ cells/well, and transfected with control and siRNAs for 24 h. We scratched the well surfaces with pipette tips along the scratch guide. Soon after scratching and after 12 h of incubation, we took images of the scratch wound using the microscope (BZ-X800; KEYENCE). Wound areas were measured by Image J and the wound recovery rate was calculated.

### Tube formation assay

96-well plates were thinly pre-coated with Matrigel Basement Membrane Matrix (#354234, Corning) in accordance with the manufacturer’s instructions. HRECs were seeded onto a 96-well plate at 2.5 × 10^4^ cells/well with EGM-2 medium containing 1% FBS and human recombinant IL-13 at 10 ng/ml. The cells were subsequently incubated for 16 h at 37 °C. The data were analyzed using Angiogenesis Analyzer macro (Gilles Carpentier, http://image.bio.methods.free.fr/ImageJ/?Angiogenesis-Analyzer-for-ImageJ) written for Image J software.

### Animals

All animal experiments were performed following the guidelines of the Association for Research in Vision and Ophthalmology (ARVO) Statement for the Use of Animals in Ophthalmic and Vision Research. The experimental protocols were approved by the Institutional Animal Care and Use Committee of Kyushu University. Wild-type C57BL/6 J mice were purchased from CLEA Japan. PN knockout (PN^−/−^) mice and TNC knockout (TNC^−/−^) mice were purchased from RIKEN BioResource Center. PN and TNC double knockout (dKO) mice line was established by crossbleeding PN^−/−^ mice with TNC^−/−^ mice.

### Mouse oxygen-induced retinopathy model

OIR mice models were generated as described previously in detail^48^. Briefly, litters of postnatal day 7 (P7) C57BL/6 J mice were exposed to 75 ± 2% oxygen for 5 days then returned to room air at P12. Mice in the control group were kept in room air from birth to P17. Mice were sacrificed by cervical dislocation and the eyes were enucleated.

### Measurement of avascular areas and neovascular tufts of OIR mice retinas

Enucleated eyeballs from OIR mice were immersed in 4% paraformaldehyde (PFA). After an hour of fixation, the neurosensory retinas were isolated from the eye cups. After an extra 24 h of immersion in the PFA, the retinas were washed by PBS and blocked by PBS containing 1% BSA and 0.5% Triton X-100 for an hour at room temperature. Then, the retinas were stained by fluorescein-labeled IB4 for 90 minutes at room temperature. The peripheral retinas were cut into petal-like shapes and mounted using the mounting medium. These retinas were examined with a fluorescence microscope (BZ-X800; KEYENCE). The images were analyzed by Image J software.

### Transfection of siRNA

HRECs were seeded onto a collagen coated 24-well plate and cultured to 50–70% confluence. PN, TNC and FN siRNAs or control siRNA (Supplementary Table) were mixed with RNA transfection reagent (Lipofectamine RNAiMAX, #13778075; Invitrogen) according to the manufacturer’s protocol. The mixtures were diluted with EGM-2 medium containing 1% FBS and the final RNA concentration was 10 ng/ml. The diluents were replaced after 12 h to the EGM-2 medium without the RNA mixtures.

### Statistical analysis

All data are presented as the mean with standard errors. The statistical significance of the differences in the measurements was determined by Wilcoxon rank sum test or Spearman’s rank correlation efficient. Differences were considered statistically significant at P < 0.05. Statistical analyses were performed using JMP software (version 13.0.0; SAS Institute).

## Supplementary information

Supplementary Information.
