# Supplementary material for: Periostin and tenascin-C interaction promotes angiogenesis in ischemic proliferative retinopathy
Source: Sci Rep. 2020 Jun 9;10:9299. doi: 10.1038/s41598-020-66278-1 (PMC7283227; doi:10.1038/s41598-020-66278-1)
Supplement: Supplementary file 1 — Supplementary Information. [file 41598_2020_66278_MOESM1_ESM.pdf]

Periostin and tenascin-C interaction promotes angiogenesis in ischemic proliferative retinopathy

Yuki Kubo[1], Keijiro Ishikawa[1*], Kenichiro Mori[1], Yoshiyuki Kobayashi[1], Takahito Nakama[1], Mitsuru Arima[1], Shintaro Nakao[1], Toshio Hisatomi[1], Masatoshi Haruta[2], Koh-Hei Sonoda[1] and Shigeo Yoshida[2]

[1]Department of Ophthalmology, Kyushu University Graduate School of Medical Sciences, Fukuoka, Japan;

[2]Department of Ophthalmology, Kurume University School of Medicine, Kurume, Japan.

*Corresponding author: Keijiro Ishikawa

Department of Ophthalmology, Kyushu University Graduate School of Medical Sciences

Phone: (092) 642-5648;

FAX: (092) 642-5663,

Email: keijishi@med.kyushu-u.ac.jp

Supplementary Information

Supplementary Figure S1

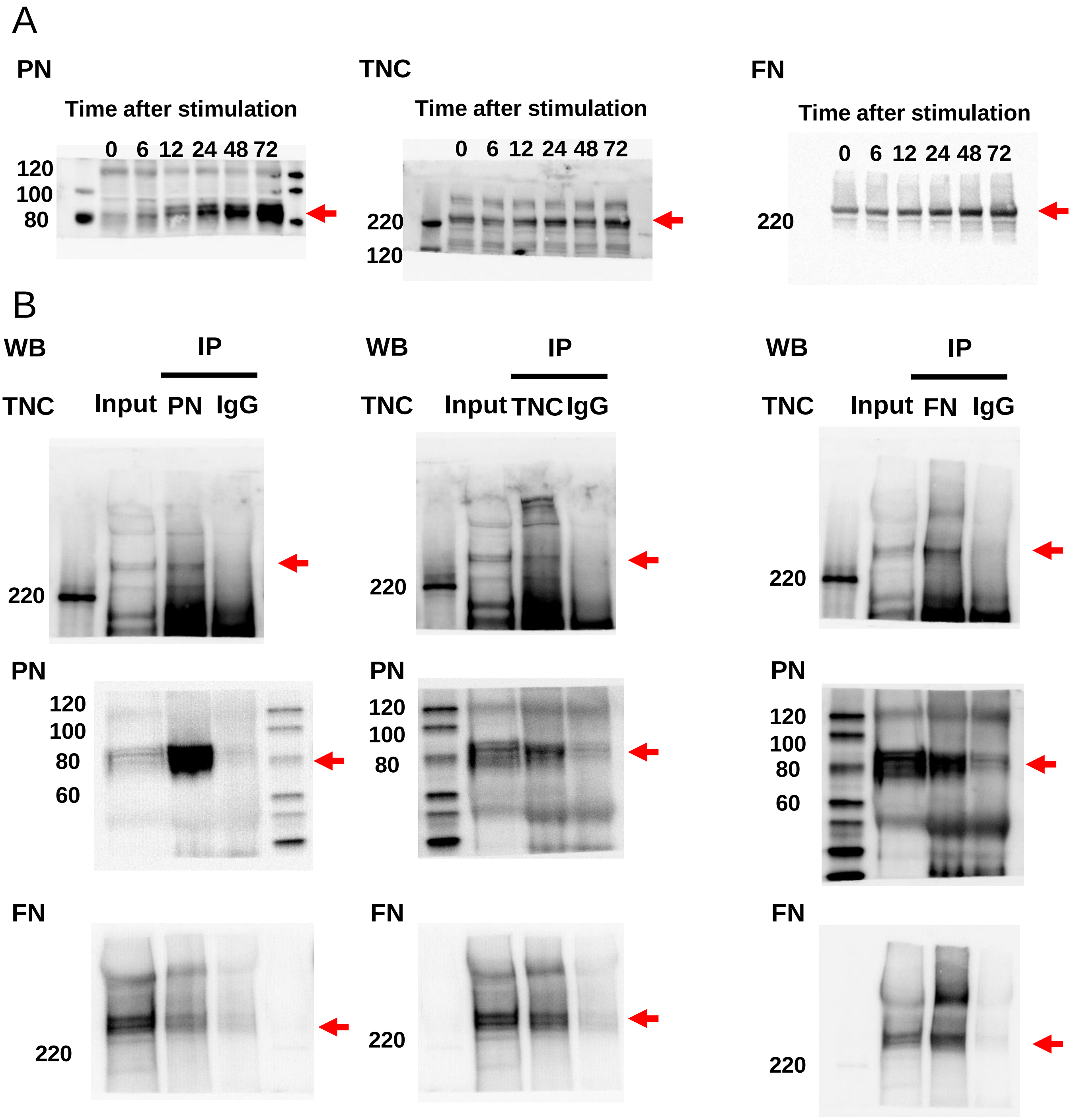

Supplementary Figure S1. Full-length gel images of Figure 2B and 2C
A: Full-length gel images of western blot analysis of PN, TNC and FN in the cell lysates of HRECs after stimulation by IL-13 at different time points. B: Full-length gel images of western blot analysis of co-immunoprecipitation assay of cell lysates from HRECs stimulated by IL-13. The cell lysates were immunoprecipitated with IgG, anti-PN antibodies, anti-TNC antibodies and anti-FN antibodies. The precipitates were analyzed by western blotting with anti-PN, anti-TNC and anti-FN antibodies. Cropped areas in the Figure 2B and 2C are lanes pointed by red arrows.

Supplementary Figure

Supplementary Figure S2

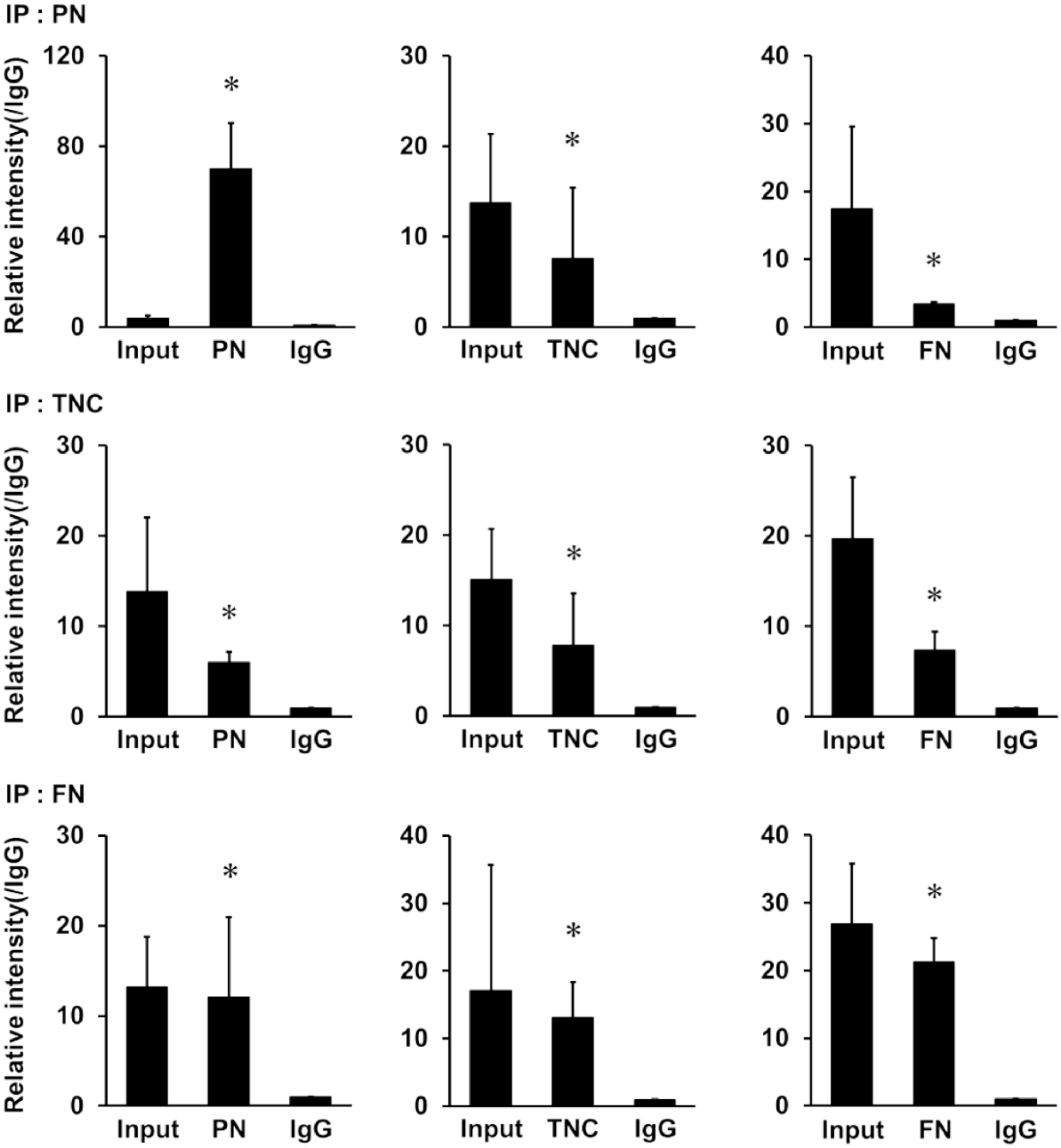

Supplementary Figure S2. Quantification of signal intensity of co-IP western blot samples. Signal intensity of co-IP samples (Figure 2C) were quantified by ImageJ software and relative intensity was calculated. Error bars represent standard deviations.

*; p<0.05, Wilcoxon rank sum test, n=4/group.

Supplementary Figure

Supplementary Figure S3

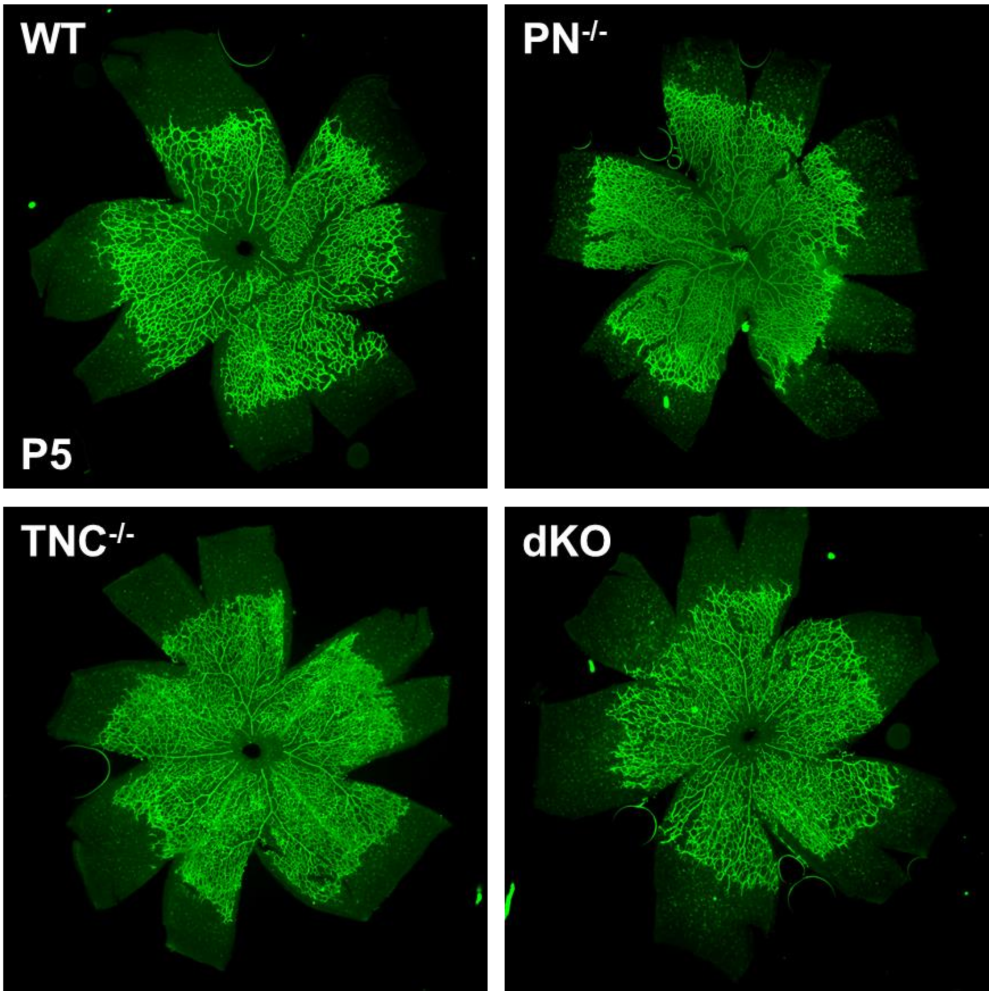

Supplementary Figure S3. Normal retinal angiogenesis in developmental stage.
This image shows normal retinal angiogenesis of WT, PN-/-, TNC-/- and dKO mice at post natal 5. There seems to be no obvious difference in normal angiogenesis between these four strain of mice.

Supplementary Figure

Supplementary Figure S4

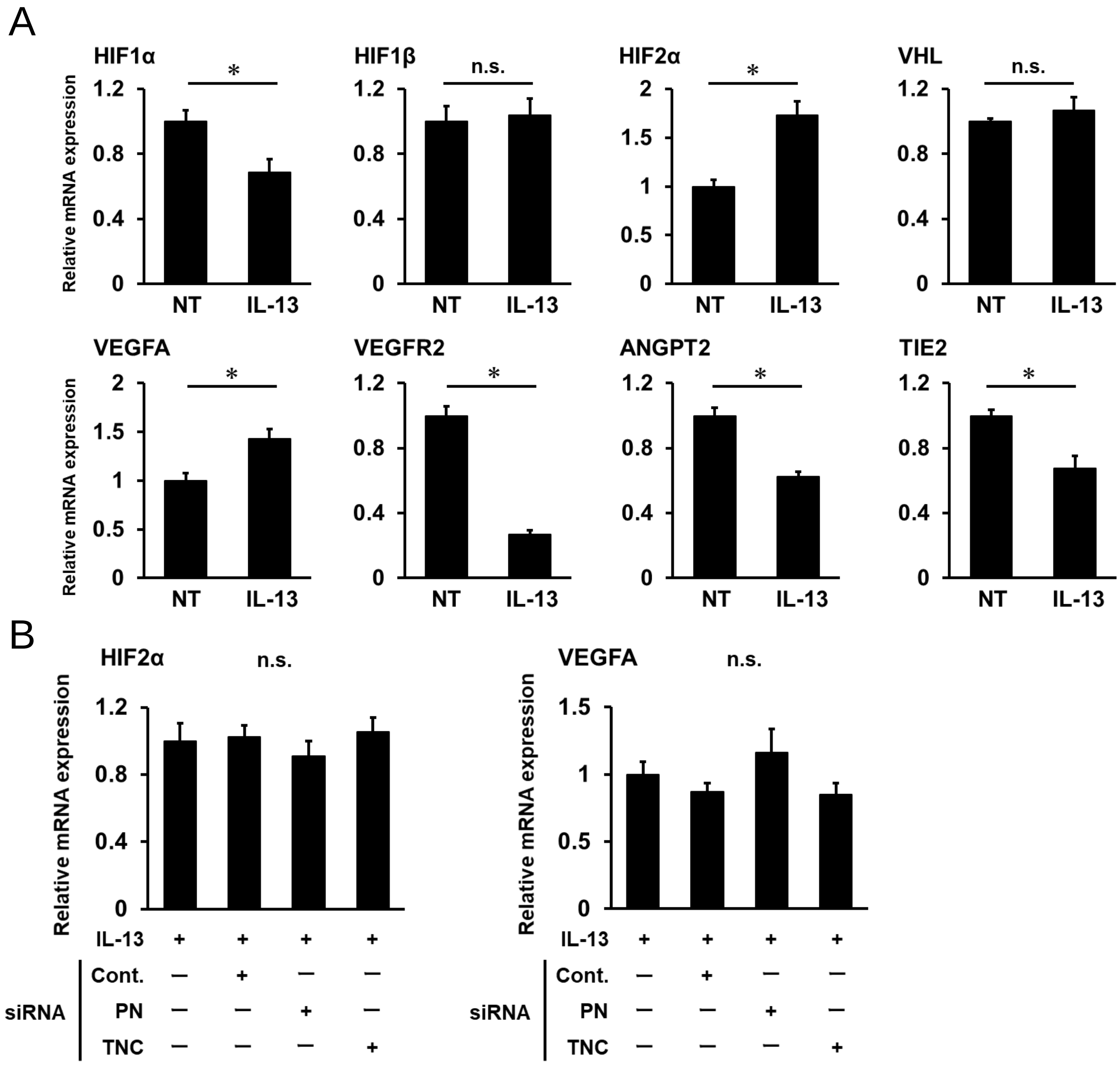

Supplementary Figure S4. Alteration of mRNA expression of HIF pathway and related genes. A: Alteration of mRNA expression of HRECs by IL-13 stimulation. HIF2α and VEGFA was significantly up-regulated. ＊; $p<0.05$, Wilcoxon rank sum test. B: Effect of PN or TNC inhibition on IL-13 induced mRNA expression of HIF2α and VEGFA. PN or TNC inhibition did not effect on the mRNA expression of HIF2α and VEGFA.

Supplementary Figure

Supplementary Figure S5

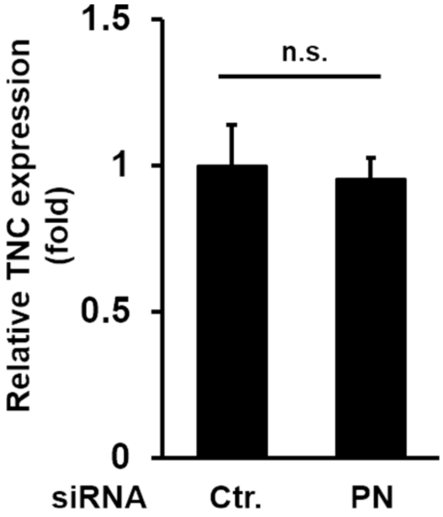

Supplementary Figure S5. TNC mRNA expression was not affected by siRNA of PN
TNC mRNA expression after IL-13 stimulation with control RNA or PN siRNA transfection determined by qRT-PCR. mRNA levels were normalized to GAPDH mRNA expression. Relative mRNA levels are shown as fold changes.
Error bars represent standard deviations. Wilcoxon rank sum test, n=4/group

Supplementary Figure S6

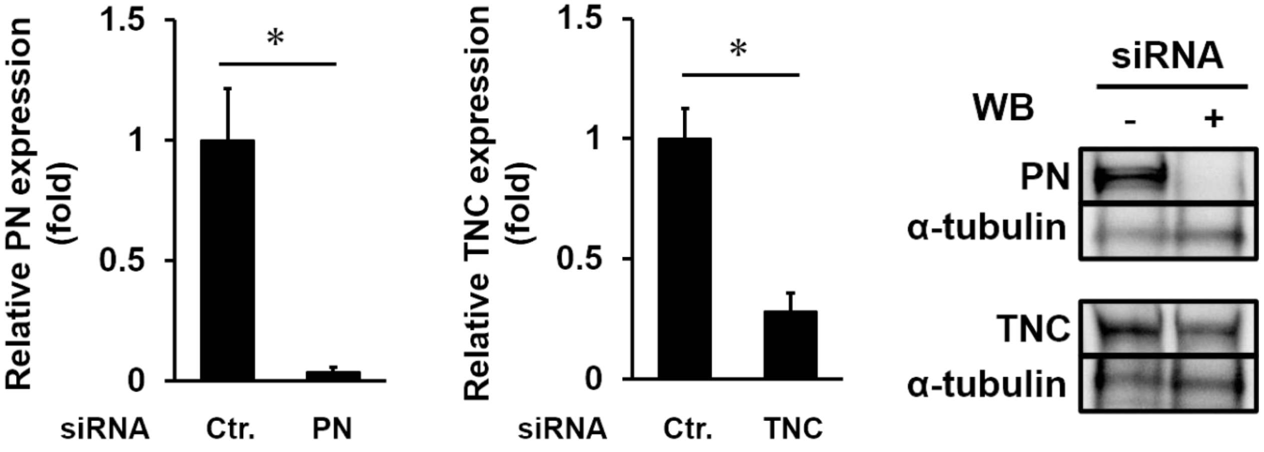

Supplementary Figure S6. Suppressive effect on PN and TNC expression by siRNA treatment. mRNA and protein expression after IL-13 stimulation with control or PN/TNC siRNA transfection. mRNA levels were determined by qRT-PCR, and were normalized to GAPDH mRNA expression. Relative mRNA levels are shown as fold changes. Error bars represent standard deviations. ＊; $p<0.05$, Wilcoxon rank sum test, n=4/group. Protein levels were determined by western blot.

Supplementary Table

ELISA kits

PN : #326070442; SHINO-TEST

TNC : #27767; IBL

FN : BMS2028; Invitrogen

Primers for SYBR green

PN : forward 5’-TGCCCAGCAGTTTTGCCCAT-3’,
reverse 5’-CGTTGCTCTCCAAACCTCTA-3’

FN : forward 5’-GAGAATAAGCTGTACCATCGCAA-3’,
reverse 5’-CGACCACATAGGAAGTCCCAG-3’

GAPDH : forward 5’-GAGTCAACGGATTTGGTCGT-3’,
reverse 5’-CTTGATTTTGGAGGGATCTCGC-3’

HIF1α : forward 5’-TATGAGCCAGAAGAACTTTTAGGC-3’
reverse 5’-CACCTCTTTTGGCAAGCATCCTG-3’

HIF1β : forward 5’-CTGCCAACCCCGAAATGACAT-3’
reverse 5’-CGCCGCTTAATAGCCCTCTG-3’

HIF2α : forward 5’-TTGCTCTGAAAACGAGTCCGA-3’
reverse 5’-GGTCACCACGGCAATGAAAC-3’

pVHL : forward 5’-GGAGCCTAGTCAAGCCTGAGA-3’
reverse 5’-CATCCGTTGATGTGCAATGCG-3’

VEGFA : forward 5’-CTTGCCTTGCTGCTCTACC-3’
reverse 5’-CACACAGGATGGCTTGAAG-3’

VEGFR2 : forward 5’-GGCCCAATAATCAGAGTGGCA-3’
reverse 5’-TGTCATTTCCGATCACTTTTGGA-3’

ANGPT2 : forward 5’-AACTTTCGGAAGAGCATGGAC-3’
reverse 5’-CGAGTCATCGTATTCGAGCGG-3’

TIE2 : forward 5’-TTAGCCAGCTTAGTTCTCTGTGG-3’
reverse 5’-AGCATCAGATACAAGAGGTAGGG-3’

Supplementary Table

Supplementary Table

TaqMan probe for qRT-PCR

TNC : Hs01115665_m1; Applied Biosystems

GAPDH : Hs02758991_g1; Applied Biosystems

Primary antibodies for immunohistochemistry and immunofluorescence

PN : AF3548; R&D systems, 10 μg/ml

(for immunohistochemistry of FVMs)

201466-T10; Sino Biological, 1/200

(for immunofluorescence of FVM sections)

50450-RP02; Sino Biological, 1/500

(for immunohistochemistry and immunofluorescence of mice OIR sections)

TNC : NB110-68136; Novus Biologicals, 1/30

FN : ab23750; abcam, 1/40

(for immunohistochemistry of FVMs and mice OIR sections

and immunofluorescence of FVM sections)

MAB88916; Millipore, 1/50

(for immunofluorescence of mice OIR sections)

CD34 : ab81289; abcam, 1/100

(for immunohistochemistry of FVMs and mice OIR sections)

RAM34; Thermo Fisher, 1/100

(for immunofluorescence of mice OIR sections)

Secondary antibodies for immunofluorescence

Anti-goat : A11056; Invitrogen, 1/1000

Anti-mouse : A21245; Invitrogen, 1/1000

Anti-rabbit : A21235; Invitrogen, 1/1000

Supplementary Table

Supplementary Table

Primary antibodies for western blotting

PN : AF3548, 1 µg/ml

TNC : NB110-68136, 5 µg/ml for lysate

#12221; CST, 1/500 for blotting of immunoprecipitation

FN : ab23750, 1 µg/ml

α-Tublin : #2125; CST, 1/1000

HRP conjugated secondary antibody for western blotting

Anti-goat : P0449; DAKO, 1/500

Anti-mouse : P0447; DAKO, 1/500

Anti-rabbit : P0448; DAKO, 1/500

Antibodies for immunoprecipitation

PN : AF3548, 25 µg/ml

TNC : #12221, 1/25

FN : ab2413; abcam, 25 µg/ml

Goat IgG : NI02; Millipore, 25 µg/ml

Rabbit IgG : NI01; Millipore, 25 µg/ml

HRP conjugated antibody for immunoprecipitation

Anti-goat : 18-8814-31; ROCKLAND, 1/1000

Anti-mouse : 18-8817-33; ROCKLAND, 1/1000

Anti-rabbit : 18-8816-33; ROCKLAND, 1/1000

siRNAs

PN : sc-61324; Santa Cruz Biotechnology

TNC : sense 5'-CUGAAAUUGGAAACUUAAATT-3',

antisense 5'- UUUAAGUUUCCAAUUUCAGTT-3'

FN : SASI_Hs01_00207242 ; Sigma-Aldrich

Supplementary Table
